# Kyste anévrismal osseux de la clavicule: à propos d’un cas

**DOI:** 10.11604/pamj.2017.27.115.11945

**Published:** 2017-06-14

**Authors:** Achraf El Bakkaly, Moulay Dris Hanine, Abdelouahed Amrani, Anouar Dendane, Sidi Zouhair Fellouss El Alami, Tarik El Madhi

**Affiliations:** 1 Service de Chirurgie Orthopédique Pédiatrique, CHU Ibn Sina / Faculté de Médecine Mohammed V, Rabat, Maroc

**Keywords:** Clavicule, exérèse radicale, kyste anévrismal, Clavicle, radical resection, anevrysmal cyst

## Abstract

Le kyste osseux anévrismal est une lésion agressive et destructrice, mais bénigne, des os longs, de la colonne vertébrale ou du bassin, touchant principalement les enfants et les jeunes adultes. Il s’agit cependant d’une véritable tumeur rare. Nous rapportons un cas d’un garçon de 8 ans,présentant une localisation très rare de kyste anévrismal, claviculaire, qui s'est initialement présenté pour une fracture sur os pathologique soit une lésion kystique bénigne. L'enfant a bénéficié d'un traitement radical avec résection complète du kyste situé au niveau de la clavicule droite avec envoi de la pièce opératoire pour étude anatomo-pathologique, confirmant notre diagnostic. L'évolution a été marquée par l'absence de récidive et un bon remodelage osseux sur le plan radiologique. Le traitement radical par résection semble permettre d'éviter la récidive. A travers notre travail, nous voulons mettre le point sur cette affection orthopédique rare en comparant nos résultats avec ceux de la littérature mondiale.

## Abstract (en)

Anevrysmal bone cyst is an aggressive, destructive but benign lesion in the long bones, spine or basin, affecting mainly children and young adults. However, it is a very rare tumor. We here report the case of a 8-year old boy with a very rare localization of anevrysmal cyst in the clavicle, initially presenting with pathologic bone fracture, that is a benign cystic lesion. The child underwent complete radical resection of the cyst located at the level of the right clavicle, followed by anatomopathological examination of surgical specimen which confirmed the diagnosis. Patient’s evolution was marked by the absence of recurrences and by good radiologic bone remodeling. Radical cyst resection seems to avoid recidivism. This study aims to update the current understanding of this rare orthopaedic condition by comparing our results with those reported in the world literature.

## Introduction

Le kyste osseux anévrismal (KOA) est une lésion particulière du squelette osseux, représente 1% des tumeurs du squelette et touche plus souvent le sujet jeune de sexe féminin [1]. La métaphyse des os longs et la colonne vertébrale en constituent les sièges préférentiels [2]. Cette lésion est définie comme une lésion pseudo-tumorale bénigne, ostéolytique expansive constituée des espaces remplis de sang et séparés par des cloisons de tissu conjonctif. C’est en 1942, que Jaffe et Lichtenstein [3] ont fait la première découverte, sous la dénomination «d’une cavité kystique gorgée de sang, soufflant l’os et séparée des parties molles par une fine coque osseuse». Nosologiquement, elle reste une lésion très controversée. Elle est aussi connue sous le terme « d’hématome sous-périosté » ou de tumeur anévrismale à cellules géantes de Ewing [4, 5]. Son étiopathogénie a suscité de nombreuses théories et sa manifestation clinique peu spécifique en rend difficile le diagnostic. L’examen anatomopathologique permet d’en affirmer la nature histologique en précisant le caractère primitif ou secondaire de la lésion. Nous rapportons une localisation exceptionnelle de kyste osseux anévrismal (KOA) situé sur la clavicule, chez un garçon de 8 ans. Notre travail va montrer l’efficacité de notre choix thérapeutique soit la résection totale du kyste pour éviter la récidive en comparant nos résultats aux ceux des autres séries de la littérature.

## Patient et observation

D.M, garçon âgé de 8ans, scolarisé, a consulté pour douleurs récidivantes de l’épaule droite avec une tuméfaction indolore en regard de l’extrémité externede la clavicule droite. L’interrogatoire retrouvait une notion de traumatisme thoracique survenu 4 mois plutôt. La tuméfaction claviculaire devenait sensible et augmentait progressivement de volume sans limitation des amplitudes de l’épaule.

**Figure 1:**
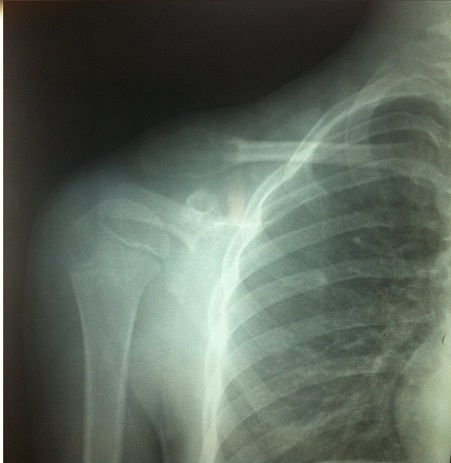
Radiographie de face de la clavicule droite montrant la lacune de l’extrémité externe

**Figure 2:**
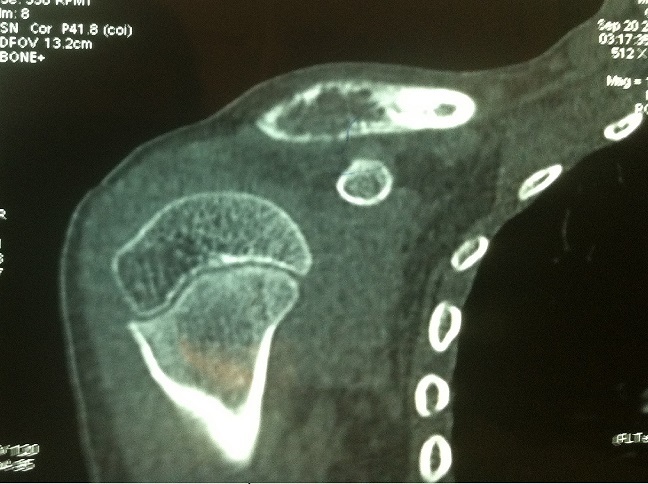
Scanner du clavicule montrant l’image lacunaire au dépend de l’extrémité externe avec important amincissement et micro-rupture de la corticale

A l’examen clinique, nous palpions une masse mesurant 6cm de diamètre dans son grand axe, peu mobile avec une peau normale en regard, non pulsatile et aucun souffle n’était observé. La radiographie standard objectivait une lacune de l’extrémité externe de la clavicule droite sans condensation périphérique ni rupture de la corticale avec intégrité des parties molles (Figure 1). Le scanner montrait une image lacunaire, soufflante au dépens de l’extrémité externe de la clavicule avec amincissement de la corticale, plus important en arrière qu’en avant. Cet amincissement est par endroits extrême, confinant la micro-rupture (Figure 2). La biopsie première a été effectuée montrant un aspect en faveur de kyste anévrismal. Ainsi, une exérèse radicale tumorale était réalisée. On retrouvait une tumeur bien circonscrite peu adhérente au plan profond avec la présence d’un réseau veineux intense afférent qui devra être parfaitement ligaturé. Cette masse d’aspect brunâtre de 2,5 cm × 4 cm présentait un contenu kystique mélangé avec du sang veineux. Cette tumeur correspondait à un stade 2 de la classification d’Enneking [6]. La fermeture était réalisée sur un drain aspiratif de Redon. Les suites opératoires ont été simples. La sortie du patient a été autorisée au deuxième jour, puis il fut régulièrement revu en consultation jusqu’à la fin de la période de rééducation. L’analyse histologique montrait une prolifération de cellules fusiformes aux noyaux réguliers et pauvres en mitoses. Ces cellules sont agencées de cellules myxoïdes avec une différenciation chondroïde périphérique et une réaction fibreuse autour. A ces éléments s’associent des cellules géantes multinucléées, des cellules xanthomateuses et des dépôts d’hémosidérine. Elle concluait à un kyste anévrismal.

**Figure 3:**
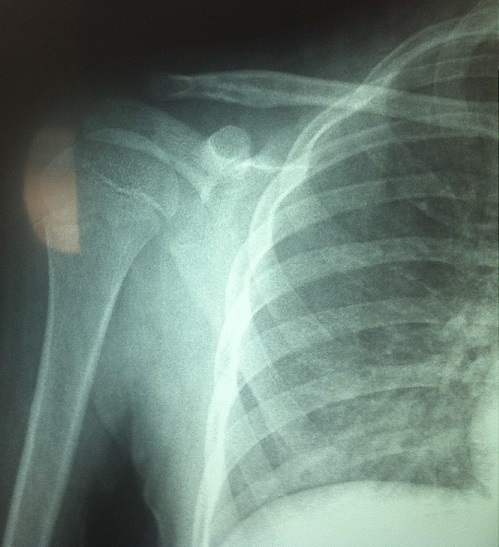
Radiographie postopératoire après un an montrant l’étendue de la résection avec un bon remodelage osseux

Après 3ans, au dernier examen, aucune complication, ni récidive n’étaient constatées (Figure 3). Les amplitudes de l’épaule apparaissaient compatibles avec les activités quotidiennes du patient.

## Discussion

Le kyste anévrysmal est une tumeur osseuse qui a été décrite pour la première fois en 1942 par Jaffe et Lichtenstein [3]. Il représente 2,5% de toutes les tumeurs osseuses. Le nom de kyste anévrysmal osseux est dû à l’aspect macroscopique de cette lésion qui est formée de cavités intra-osseuses remplies de sang mais cette lésion n’est ni un kyste (car la cavité osseuse ne possède pas un revêtement épithélial propre et le contenu n’est pas toujours liquidien puisqu’il peut s’agir de tissu osseux dystrophique), ni un anévrisme (car le contenu hématique n’est pas à l’intérieur de vaisseaux) [6, 7]. L’incidence de cette affection est de 0,14 pour 100 000 habitants [4]. Les sièges les plus fréquents de KOA sont la métaphyse des os longs des membres inférieurs (40,80 %) en particulier le tibia (17,85 %), le fémur (15,94 %) et le bassin [1]. La localisation d’un KOA à la clavicule a été peu rapportée [8–10] et ne concernerait, selon Wilner [11], qu’environ 3 % de cette entité pseudotumorale. La rareté de ces lésions présente un défi pour la plupart des chirurgiens orthopédistes, d´où l’intérêt des connaissances sur les lésions claviculaires aidera à la gestion opportune et appropriée. Notre observation concerne un garçon de huit ans,80 % des cas de KOA sont rapportés ont un âge inférieur à 20 ans [12] avec une prédominance pour la première décade faisant penser qu’il survient essentiellement dans le squelette immature. Ce n’est pourtant pas une entité exclusive de l’enfance et de l’adolescence puisque ça peut apparaître chez des adultes [13]. Le diagnostic différentiel de kyste osseux anévrysmal comprend les tumeurs à cellules géantes, fibrome chondromyxoïde et ostéosarcome télangiectasique [1]. Le kyste osseux anévrysmal a des caractéristiques radiologiques particulières et distinctives [14]. Il faut souvent un pathologiste habitué aux tumeurs osseuses pour en établir le diagnostic histologique formel.

Bien que l’étiopathogénie reste encore discutée. Il est probable que le kyste osseux anévrismal résulted’une perturbation hémodynamique locale avec augmentation de la pression veineuse ou création de communication artério-veineuse anormale [3]. Pour Campanacci et al. [15], il s’agirait d’une réaction tissulaire liée à une hémorragie locale. Les études cytogénétiques n’ont pas apporté plus d’information [16, 17]. Szendroi et al [12], à partir d’une étude angiographique et immunohistochimique, ont montré la présence de vaisseaux efférents tortueux et dilatés responsables d’une augmentation des résistances veineuses périphériques. Devant ladécouverte de KOA diaphysaire, Certains auteurs ont avancé une hypothèse fondée sur un asynchronisme de la circulation sanguine entraînant une opposition du flux sanguin entre réseau vasculaire médullaire et périoste aboutissant à une élévation de la pression locale [18]. En l’absence d’étude vasculaire dans notre cas, il serait difficile de proposer une hypothèse étiopathogénique formelle. Cependant, la notion de traumatisme existait et la proximité du réseau veineux sous-clavier au milieu de la clavicule pouvait expliquer la possibilitéde perturbation hémodynamique de cette veine dont les nombreuses afférences avec la face inférieure de la clavicule auraient pu développer le KOA et être ainsi à l’origine de l’ostéolyse métaphysaire. Comme le soulignent Anoumou et ses collaborateurs [18], les connaissances sur la pathogénie des KOA ne se sont pas améliorées depuis Lichtenstein. D’une manière générale la symptomatologie des KOA est non spécifique. Dans notre cas, la tuméfaction avait rapidement augmenté de volume sur une période de quatre mois avec l’installation secondaire de douleurs qui restaient épisodiques. La lésion devient parfois localement agressive et les fractures pathologiques constituent un mode fréquent de révélation [8, 19]. Les douleurs kystiques sont inexpressives, sourdes, leur intensité augmente avec la présence de fractures pathologiques et il faut classiquement huit mois pour établir le diagnostic [13]. Dans notre cas, le diagnostic a été établi au bout de six mois d’évolution. L’image radiographique classique est une image lacunaire ou multilacunaire soufflant les limites de l’os. L’étude radiographique permet de distinguer trois phases évolutives. Notre cas correspond au stade 2 d’Enneking [20], dit phase intermédiaire ou active car le kyste a atteint un volume considérable soufflant et déformant la pièce osseuse.Le scanner et l’IRM sont utiles à la fois pour faire le diagnostic et pour la détermination de l´étendue de la tumeur.

La tomodensitométrie est particulièrement utile pour délimiter le kyste lorsqu’il est situé dans des localisations particulières, comme la colonne vertébrale ou au niveau du bassin. Elle est d’un apport considérable, en montrant le niveau liquidien résultant de la sédimentation entre le sang frais et le sang vieilli. Les images TDM ne sont pas pathognomoniques des KOA. En ce qui concerne la clavicule, la situation anatomique en « S allongé » étalé entre le sternum et l’omoplate rend difficile l’appréciation des images. La réalisation d’image 3 D paraît pourtant primordiale pour bien identifier la lésion. Dans notre cas le scanner 2 D réalisé n’a pas permis une orientation rapide du diagnostic. Pour Vedantam et al. [10], la reconstruction 3D de la clavicule précise au mieux les limites de la tumeur. L’IRM dans la littérature apporte un complément d’arguments mais n’a pas été réalisée dans notre cas.La résonance magnétique du KOA est caractérisée par quatre signes évocateurs : un hypersignal tumoral T2, une limite tumorale nette, un liséré périphérique de bas signal, et de nombreuses logettes qui délimitent les septa [7, 14, 21]. L’examen radiologique apporte un diagnostic de probabilitéqui doit toujours être confirmé par une analyse anatomopathologique.L’étude histologique permet ainsi de préciser la nature de la tumeur et d’affirmer ou d’éliminer la présence des lésions associées. Des cellules géantes sont souvent retrouvées, elles diffèrent, selon Server et al. [13], de celles observées dans les tumeurs à cellules géantes (cellules plus petites, et noyaux moins nombreux). La présence de cellules géantes confirme le caractère actif de la tumeur [1]. Elle permet ainsi d’éliminer l’existence d’une autre tumeur dans le cadre d’un KOA secondaire [16]. Cette forme secondaire serait réactionnelle et associée à une lésion préexistante qu’il peut totalement détruire. Les lésions associées peuvent être bénignes (tumeur à cellules géantes, chondroblastome, fibrome chondromyxoïde, ostéoblastome, dysplasie fibreuse, fibrome non ossifiant, kyste osseux essentiel) ou parfois malignes (ostéosarcome télangiectasique, chondrosarcome à cellules claires, et hémangioendothéliome) [15].

Malgré la rareté des KOA, les essais thérapeutiques les concernant connaissent en revanche un essor constant. L’utilisation plus récente de protéine ostéoinductrice [22] et d’injection de calcitonine [23] semble être une voie prometteuse. Schreuder et al. [2] dans une revue de la littérature précise le pourcentage de récidive en fonction du traitement. Ainsi, aucune récidive n’est observée après résection large, alors que 30,8 % de récidives étaient constatés après curetage simple. Dans notre cas nous avons réalisé une exérèse radicale du kyste. Le volume de la tumeur et de la lyse importante n’autorisait pas un traitement conservateur. Aucune récidive n’est notée au dernier examen. Cottalorda et al. [7] conseillent d’étendre cette indication de résection large sur les os grêles comme le péroné et les côtes.

Dans la littérature toutes les localisations claviculaires de KOA ont été traitées par exérèse radicale [8–10]. La radiothérapie, en raison du risque de sarcome induit garde peu d’indication et les embolisations doivent être réalisées par des mains entraînées, sans en occulter les risques d’embolies pulmonaires ou d’ischémie des structures vitales et neurologiques. La proximité de la clavicule par rapport aux vaisseaux et au dôme pleuralconstitue une limite à ces techniques. La cryochirurgie à l’azote liquide et l’utilisation d’éthibloc sont des moyens connus mais le faible recul ne permet pas encore une utilisation étendue.

## Conclusion

Le kyste osseux anévrysmal est une lésion particulière, survenant principalement dans les deux premières décennies de vie, rapidement progressive, d’aspect impressionnant à l’imagerie, mais dont le pronostic est globalement bon. La confirmation du diagnostic est cependant histologique. Le traitement radical par résection donne satisfaction : pas de récidive et bon remodelage osseux ensuite radiologiquement.

## Conflits d’intérêts

Les auteurs ne déclarent aucun conflit d’intérêt.
